# A Method Combining Discrete Cosine Transform with Attention for Multi-Temporal Remote Sensing Image Matching

**DOI:** 10.3390/s25051345

**Published:** 2025-02-22

**Authors:** Qinyan Zeng, Bin Hui, Zhaoji Liu, Zheng Xu, Miao He

**Affiliations:** 1 Key Laboratory of Opto-Electronic Information Processing, Chinese Academy of Sciences, Shenyang 110016, China; 2 Shenyang Institute of Automation, Chinese Academy of Sciences, Shenyang 110016, China; 3 University of Chinese Academy of Sciences, Beijing 100049, China

**Keywords:** image matching, remote sensing, discrete cosine transformation, channel attention, sparse attention

## Abstract

Multi-temporal remote sensing image matching is crucial for tasks such as drone positioning under satellite-denial conditions, natural disaster monitoring, and land-cover-change detection. However, the significant differences between multi-temporal images often lead to the reduced accuracy or even failure of most image matching methods in these scenarios. To address this challenge, this paper introduces a Discrete Cosine Transform (DCT) for frequency analysis tailored to the characteristics of remote sensing images, and proposes a network that combines the DCT with attention mechanisms for multi-scale feature matching. First, DCT-enhanced channel attention is embedded in the multi-scale feature extraction module to capture richer ground object information. Second, in coarse-scale feature matching, DCT-guided sparse attention is proposed for feature enhancement, which suppresses the impact of temporal differences on matching while making the amount of computation controllable. The coarse-scale matching results are further refined in the fine-scale feature map to obtain the final matches. Our method achieved correct keypoint percentages of 81.92% and 88.48%, with average corner errors of 4.27 and 2.98 pixels on the DSIFN dataset and LEVIR-CD dataset, respectively, while maintaining a high inference speed. The experimental results demonstrate that our method outperformed the state-of-art methods in terms of both robustness and efficiency in the multi-temporal scenarios.

## 1. Introduction

Multi-temporal remote sensing image matching is a critical task in geospatial analysis, facilitating applications such as environmental monitoring, urban development tracking, and disaster assessment. The accurate alignment of images captured at different times is essential for detecting changes and ensuring the integrity of subsequent analyses. Traditional image matching methods often rely on feature detection and description [1,2]. However, maintaining robust correspondences becomes challenging in the presence of significant temporal variations, such as different sensors, seasonal effects, and changes in urban structures.

Recent advances in deep learning have introduced sophisticated image matching frameworks, notably SuperGlue [3] and LoFTR [4]. SuperGlue introduces an innovative application of self-attention and cross-attention mechanisms for feature matching. LoFTR builds on this application of attention mechanisms and further extends this approach by combining a pixel-wise dense matching strategy and a coarse-to-fine matching method. Although these methods have demonstrated impressive performances in general image matching tasks, their effectiveness in multi-temporal remote sensing applications is limited due to the significant appearance variations inherent in such data.

To address these limitations, this paper presents an enhanced image matching method based on the LoFTR framework that was specifically designed for multi-temporal remote sensing images. Our approach integrates Discrete Cosine Transform (DCT)-based attention mechanisms to leverage frequency domain information, which is rich in the structural and textural patterns crucial for distinguishing different land types and monitoring temporal changes. By incorporating the DCT into both the feature extraction and matching stages, our method enhances feature discrimination and reduces computational complexity, ultimately improving the matching accuracy and efficiency.

The proposed methodology introduces two key innovations. On the one hand, DCT-based channel attention modules are integrated into the CNN backbone of LoFTR, enabling the network to leverage frequency information to emphasize salient features. Different land covers in remote sensing images often have unique frequency distributions [5]. We exploited this character to innovatively introduce frequency information into the feature extraction process. By utilizing frequency information to generate channel attention, we enhanced the robustness and discrimination of feature representations, especially when dealing with significant appearance changes in multi-temporal remote sensing images. On the other hand, a DCT-guided sparse attention mechanism was introduced in the coarse matching stage that was inspired by DCT’s application in image compression. By innovatively integrating DCT with a sparse attention mechanism, we extracted frequency information to generate attention guides. This approach allowed us to perform self-attention or cross-attention calculations on the most relevant windows, minimizing interference from temporal difference regions in the attention mechanism and enhancing the computational efficiency. Through extensive experiments on diverse multi-temporal remote sensing datasets, we demonstrated that integrating DCT-based attention mechanisms into the LoFTR framework significantly enhanced both the matching accuracy and computational efficiency compared with existing image matching methods.

In summary, the main contributions of this paper are as follows:

1. We introduce a novel method for integrating DCT-based frequency channel attention into the CNN backbone, enhancing the feature robustness and discrimination in multi-temporal remote sensing scenarios.
2. We propose a frequency-guided sparse attention mechanism to enhance coarse-scale features. By narrowing the attention scope, this module minimizes noise introduced by the temporal difference region and simultaneously reduces the computational complexity.
3. Through comprehensive experiments, we validated that our DCT-integrated approach outperformed existing image matching frameworks in terms of the robustness and efficiency on multi-temporal remote sensing datasets.

## 2. Related Works

### 2.1. Image Matching Methods for Multi-Temporal Remote Sensing Images

Remote sensing image matching methods can be broadly categorized into area-based methods and feature-based methods [6,7,8,9]. Feature-based methods extract local invariant features for matching, which is more resistant to temporal changes and is therefore more suitable for multi-temporal scenarios. Traditional feature-based matching methods typically involve first detecting features, followed by a feature description [2,10,11,12]. Rasmy et al. [13] proposed a method combining Fourier phase correlation and the Harris detector in order to achieve a sub-pixel registration accuracy. Aiming to improve the temporal robustness, OFM [14] uses multioriented filters to convolute images and extract the orientation indices to construct the feature maps. However, this type of manual design method relies on prior knowledge and is only applicable to multi-temporal scenes with small changes in ground objects. The introduction of deep learning can bring significant performance improvements, especially in scene robustness. Yang et al. [15] used a pre-trained VGG network to generate robust multi-scale feature descriptors. Liu et al. [16] used spatial multi-scale convolutional layers to construct a Siamese network to align multi-temporal features. These methods still follow the framework of feature detection followed by feature description. However, the complex temporal variations make it challenging to reliably detect keypoints. Recently, detector-free image matching methods [17,18,19], designed for general tasks, have emerged, offering new solutions to this problem. NBR-Net [20] used the bidirectional estimation of pixel flow to enhance the multi-temporal image matching consistency, but its performance remained constrained by the limited receptive field of CNNs. SC-Net [21] used an attention mechanism to fuse local features and global features of a single image to improve the recognition of repeated scenes, but it still lacks the ability to resist obvious temporal differences.

### 2.2. Deep Learning-Based Image Matching Methods for General Tasks

With the rise of deep learning, researchers have increasingly utilized deep learning models to autonomously extract image features, replacing traditional low-level image information with high-level features learned. Early image matching methods mainly employed Siamese networks, where two sub-networks with shared weights were used to extract meaningful features and then describe them [22,23,24,25]. Innovatively, SuperPoint [26] proposed a dual-branch network framework for self-supervised training, enabling simultaneous keypoint detection and descriptor generation. D2-Net [27] further developed this idea, transforming convolutional networks into dense feature descriptors and detectors. NCNet [17] adopted a detector-free approach to directly learn dense correspondences in an end-to-end manner. In recent years, Transformer [28] was introduced into computer vision [29]. To handle large viewpoint variations, SuperGlue [3] used a Transformer to expand the receptive field of keypoints. LoFTR [4] combined the Transformer with the detector-free strategy to solve the matching problem in low-texture scenes. The framework of LoFTR has inspired numerous subsequent works [30,31,32]. However, in multi-temporal scenarios, the challenge is not the lack of texture or the large change in viewpoint, but rather the interference caused by temporal variations that disrupt the texture consistency. These variations introduce significant noise and distortions, which are often difficult to differentiate from the invariant features, presenting a unique challenge compared with general tasks.

### 2.3. DCT in Deep Learning

Common methods for space–frequency domain conversion are the Discrete Fourier Transform (DFT) and the DCT. The DFT uses complex numbers to represent frequency domain information, which introduces additional computational complexity. In contrast, the DCT uses real numbers. It is computationally efficient and has good energy concentration, so it is often used for image denoising [33,34,35] and image compression [36,37,38]. In recent years, the DCT was introduced into deep learning as an efficient feature extraction tool [39,40,41]. Ghosh et al. [42] believed that the combination of DCT and convolutional networks can generate sparser weight matrices. FCANet [43] first used the DCT to generate a channel attention mechanism that achieved excellent performance in multiple tasks. Chaudhury et al. [44] performed a DCT transformation on the input and found that the network trained in the cosine domain is more resistant to noise than that trained in the spatial domain. These works inspired us to integrate the DCT into our method.

## 3. Method

**Figure 1:**
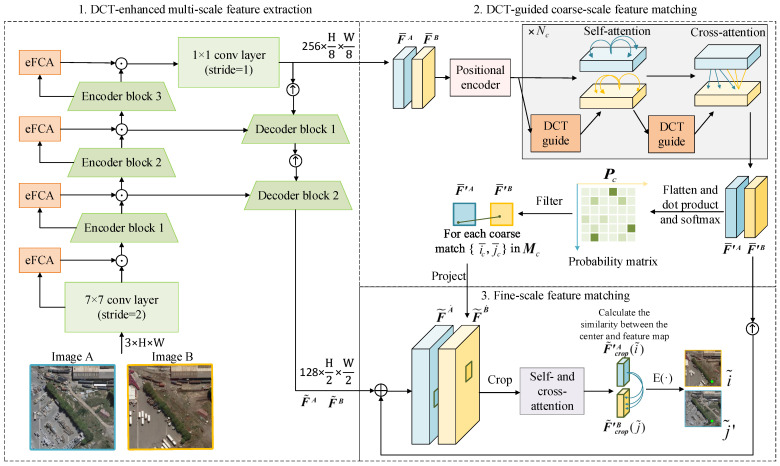
Overall framework of the proposed method.

Our method can be divided into three stages: DCT-enhanced multi-scale feature extraction, DCT-guided coarse-scale feature matching, and fine-scale feature matching. The overall structure is shown in Figure 1.

### 3.1. DCT-Enhanced Multi-Scale Feature Extraction

**Figure 2:**
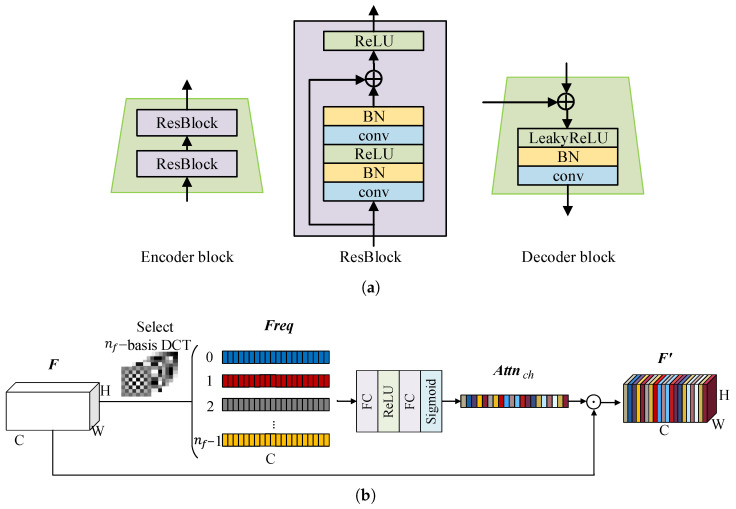
The details in the feature extraction module. (**a**) The structure of the blocks. BN is short for Batch Normalization. (**b**) The structure of the enhanced frequency channel attention (eFCA).

Given a pair of images A and B to be matched, they are input into a multi-scale feature extraction module that integrates a feature pyramid network (FPN) [45] with enhanced frequency channel attention (eFCA). This module extracts deep features from the images and ultimately outputs both coarse-scale feature maps ${\bar{F}}^{A}$ and ${\bar{F}}^{B}$ and fine-scale feature maps ${\tilde{F}}^{A}$ and ${\tilde{F}}^{B}$. The overall framework of the module is shown in Figure 1 and more details are in Figure 2.

Remote sensing images usually contain a wealth of information, such as the topography, vegetation cover, and water distribution. An FPN enhances the model’s ability to handle scenes with large scale differences in remote sensing images through multi-scale feature fusion. However, the downsampling operation in an FPN reduces the image resolution, leading to a loss of some fine details that may be essential [46]. This presents a significant challenge for extracting abundant features from remote sensing images. Frequency domain analysis can capture information that is difficult to distinguish in the spatial domain by separating different frequency components within the image. For example, the high-frequency components typically contain detailed features, such as edges and lines, which have high stability and recognizability. Additionally, frequency domain analysis aids in distinguishing various land-cover types, thereby enhancing the ability to perceive changes in land objects. Therefore, before downsampling the feature map, we used frequency domain information to generate the eFCA to assist the network in adjusting the saliency of different channels adaptively. This module enhances the representation of key information in the feature map and suppresses irrelevant interference, ensuring that the downsampled feature map retains as many critical details as possible.

For the feature extraction stage, we focused on the structural and textural features of the image, which are mainly reflected in the amplitude of the image frequency domain representation. To ensure computational efficiency and maintain the ability to perceive image information, we chose the DCT rather than the DFT.

Assuming the image f(x,y) size is M×N, Two-Dimensional (2D) DCT can be expressed as

(1) $$F\left(u,v\right)=\alpha \left(u\right)\alpha \left(v\right)\sum_{x=0}^{M-1}\sum_{y=0}^{N-1}f\left(x,y\right)\cos\left[\frac{\pi (2x+1)u}{2M}\right]\cos\left[\frac{\pi (2y+1)v}{2N}\right]$$

where *u* and *v* are the 2D frequency components corresponding to f(x,y), while α(u) and α(v) are normalizing constants. α(u) and α(v) have the following values:

(2) $$\alpha (u)=\left\{\begin{array}{c} \sqrt{\frac{1}{M}},u=0\\ \sqrt{\frac{2}{M}},u\neq 0 \end{array},\alpha (v)=\left\{\begin{array}{c} \sqrt{\frac{1}{N}},v=0\\ \sqrt{\frac{2}{N}},v\neq 0 \end{array}\right.\right.$$

Inspired by FCANet [43], we designed an enhanced frequency channel attention (eFCA) module, as shown in Figure 2. In FCANet, the features are divided into several parts evenly along the channel dimension. Each part of the features uses only one specific set of frequency components to obtain the channel attention. This strategy of uniformly grouping features and considering only a single frequency component limits the integrity of the information. To address this limitation, we improved the approach by using multiple sets of frequency components to compute feature maps for all channels. We then integrated different frequency components through linear mapping to generate the channel attention. Thus, more comprehensive frequency information is introduced in the eFCA, enabling the channel attention to better capture the diversity and complexity of the feature details. The experimental comparison of the channel attention is shown in detail in Section 4.4.

Further considering the computational complexity, it is impractical to calculate all the M×N frequency components for each feature map. To ensure the stability of the results during training and inference, it is necessary to maintain consistent relative frequency positions across the DCTs of varying sizes. Therefore, in the implementation, a predefined list of $n_{f}$ groups of *u* and *v* values is established for a 7×7 DCT size. For DCT transforms of different sizes, corresponding scaling is performed to ensure the consistency of relative frequency positions. This can effectively incorporate important frequency information into the decision-making process of the model while avoiding too many redundant calculations or too much noise. The model is able to adaptively adjust the weights of these frequency components through learning according to different image features, giving priority to those frequency components that are conducive to matching.

Suppose $F\in R^{C\times H\times W}$ is the feature map in the networks, and $u_{i}$, $v_{i}$ ($i\in [0,n_{f}-1],i\in Z$) is one set of frequency components from the *u* and *v* lists. The DCT coefficients of these frequencies corresponding to the *j*-th (j∈[0,C−1],j∈Z) channel feature can be calculated using the above Equation (1). Therefore, the DCT coefficient of *F* at $\left[u_{i},v_{i}\right]$ can be expressed as

(3) $${\mathrm{Freq}}_{i}={\mathrm{DCT}}_{\left[u_{i},v_{i}\right]}\left(F\right)$$

After obtaining ${\mathrm{Freq}}_{i}\in R^{1\times C}$, F is calculated at all the selected $n_{f}$ groups of frequency components to obtain $\mathrm{Freq}\in R^{n_{f}\times C}$. The channel attention ${\mathrm{Attn}}_{\mathrm{ch}}\in R^{C}$ is obtained through linear mapping, which can be written as

(4) $${\mathrm{Attn}}_{\mathrm{ch}}=\mathrm{Sigmoid}\left(\mathrm{FC}\left(\mathrm{ReLU}\left(\mathrm{FC}\left(\mathrm{Freq}\right)\right)\right)\right)$$

Each channel of F is scaled by the corresponding attention value:

(5) $$F^{\prime }={\mathrm{Attn}}_{\mathrm{ch}}⊙F$$

Through the above process, the eFCA integrates the rich frequency domain information from the feature map and assists the FPN in adjusting the saliency of different channels through learning, thereby preserving important features during convolutional downsampling.

### 3.2. DCT-Guided Coarse-Scale Matching

Before matching, we used sine and cosine functions of different frequencies to perform 2D position encoding [47] on the feature maps ${\bar{F}}^{A}$ and ${\bar{F}}^{B}$ obtained from the backbone. We then applied DCT-guided sparse attention for self-attention and cross-attention to further enhance both of them.

The advantages of attention-mechanism-enhanced features were demonstrated in numerous image matching works [48,49,50]. The input vectors of the attention layer are typically called the query (Q), key (K), and value (V). The attention operation computes the similarity between Q and each K to assign weights to the corresponding V, with the final output being the weighted sum of V. However, remote sensing images often have a high resolution and large size. Even at a coarse scale, the computational burden imposed by the vanilla attention [28] is still unbearable. Moreover, attention interactions involve many redundant calculations. Not all features are helpful for updating the current features. For example, interactions with regions with large temporal differences exhibiting large temporal differences can introduce unnecessary noise, hindering the effective update of current features.

**Figure 3:**
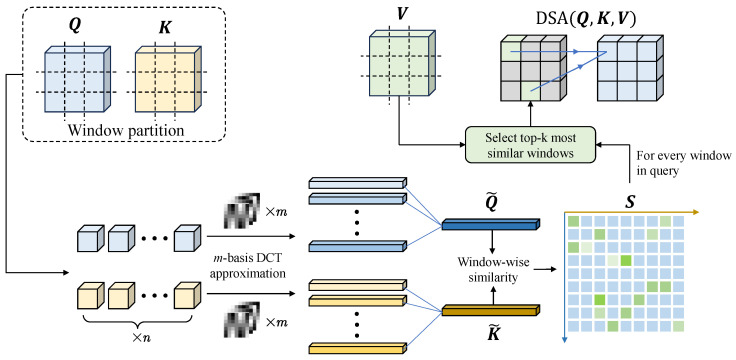
The flowchart of DCT-guided sparse attention. For self-attention, Q, K, and V all come from the feature map to be updated. For cross-attention, Q comes from the feature map to be updated, while K and V come from the other one.

To address these issues, this paper proposes a DCT-guided sparse attention (DSA) mechanism, which aims to use the frequency information of the feature map to measure the relevance and similarity between the current region and the other regions, generating attention guides that focus the attention on the most relevant features, thus reducing the scope of attention interaction and alleviating the computational burden. The reason DCT is chosen again here is that it concentrates the image’s energy in the low-frequency components, which are sufficient to represent the general information of the image. In this stage, this advantage enables DCT to effectively represent the window content, as it captures the approximate features of the window by analyzing only the low-frequency components so as to effectively assess the similarity. The flowchart of the DSA is shown as Figure 3. We took self-attention as an example to explain the DSA in detail.

Given a feature map $X\in R^{H\times W\times C}$, it is first partitioned into *n* non-overlapping windows, each of size $w_{c}\times w_{c}$, where

$$n=\left\lfloor \frac{H}{w_{c}}\right\rfloor \times \left\lfloor \frac{W}{w_{c}}\right\rfloor$$

The query, key, and value representations for attention are obtained through linear projections:

(6) $$Q=XW_{Q},\;K=XW_{K},\;V=XW_{V}$$

where $W_{Q},W_{K},W_{V}\in R^{C\times d}$ are learnable projection matrices.

As shown in Figure 3, we first applied the 2D DCT to each window to compress it spatially:

(7) $$\begin{array}{cc} {\hat{Q}}_{i} & ={\mathrm{DCT}}_{m}\left(Q_{i}\right), \end{array}$$

(8) $$\begin{array}{cc} {\hat{K}}_{i} & ={\mathrm{DCT}}_{m}\left(K_{i}\right)\;\mathrm{for}\;i=1,\ldots ,n \end{array}$$

where ${\mathrm{DCT}}_{m}\left(\cdot \right)$ denotes the operation that retains *m* frequency components after the 2D DCT transformation [43]. This compression results in ${\hat{Q}}_{i},{\hat{K}}_{i}\in R^{m\times d}$ for the *i*-th window.

The compressed representations are then projected using a learnable projection matrix:

(9) $$\tilde{Q}=\hat{Q}W_{P},\;\tilde{K}=\hat{K}W_{P}$$

where $W_{P}\in R^{m\times 1}$ is the projection matrix, resulting in $\tilde{Q},\tilde{K}\in R^{n\times d}$.

The similarity matrix $S\in R^{n\times n}$ between windows is computed using

(10) $$S=\frac{\tilde{Q}{\tilde{K}}^{⊤}}{\sqrt{d}}$$

For each query window *i*, the top-*k* most similar key and value windows are selected:

(11) $$\Omega_{i}={\mathrm{top}}_{k}\left(S_{i,:}\right)$$

For each pixel *p* in the *i*-th query window, its attention is computed with all pixels in the selected *k* key and value windows:

(12) $${\mathrm{DSA}}_{i}^{p}\left(Q,K,V\right)=\sum_{j\in \Omega_{i}}\sum_{q\in P}\mathrm{softmax}\left(\frac{Q_{i}^{p}{\left(K_{j}^{q}\right)}^{⊤}}{\sqrt{d}}\right)V_{j}^{q}$$

where p,q∈P denote pixel positions within a window, $Q_{i}^{p}\in R^{1\times d}$ is the query feature at position *p* in the *i*-th window, and $K_{j}^{q},V_{j}^{q}\in R^{1\times d}$ are the key and value features at position *q* in the *j*-th window. This results in a sparse attention pattern where each query window (comprising $w_{c}\times w_{c}$ pixels) attends to *k* selected key and value windows (a total of $k\times w_{c}\times w_{c}$ pixels).

Using DCT guidance, the attention is made sparse, which has a significant effect on reducing the amount of computation. The specific comparison of the computational complexities of vanilla attention and DSA are presented as follows:

1. Vanilla attention:
  - Each query pixel attends to all H×W pixels.
  - Feature dimension: *d*.
  - Computational complexity: $O\left({\left(HW\right)}^{2}d\right)$.
2. Our DCT-guided sparse attention (DSA):
  - Each query pixel attends to *k* windows, each containing w×w pixels.
  - Total number of windows: $n=\frac{HW}{w^{2}}$.
  - Selected windows per query: k≪n.
  - Pixels per window: $w^{2}$.
  - Feature dimension: *d*.
  - Number of DCT basis (constant): *m*.
  - Computational complexity:
    – DCT compression: $O\left(mHWd\right)$.
    – Window similarity calculation: $O\left(n^{2}d\right)$.
    – Attention calculation:
      $$O\left(nw^{2}\cdot kw^{2}d\right)=O\left(\frac{HW}{w^{2}}\cdot w^{2}\cdot kw^{2}d\right)=O\left(HWkw^{2}d\right).$$
    – Total:
      $$O\left(mHWd+n^{2}d+HWkw^{2}d\right)=O\left((n^{2}+HW\left(m+kw^{2}\right))d\right).$$

Therefore, the computational reduction ratio between the vanilla attention and the DSA can be calculated by

$$\mathrm{Reduction}\;\mathrm{Ratio}=\frac{{\left(HW\right)}^{2}d}{(n^{2}+HW\left(m+kw^{2}\right))d}=\frac{{\left(HW\right)}^{2}}{n^{2}+HW\left(m+kw^{2}\right)}$$

For a typical setting of our method:

- Image size: H=W=64.
- Window size: w=8.
- Selected windows: k=8.
- DCT basis: m=4.

The computational reduction ratio between the vanilla attention and the DSA can be calculated by

$$\mathrm{Reduction}\;\mathrm{Ratio}=\frac{{\left(64\times 64\right)}^{2}}{64^{2}+64\times 64\times \left(8\times 8^{2}+4\right)}\approx 8.$$

We employed the DSA introduced above to perform alternating self-attention and cross-attention on ${\bar{F}}^{A}$ and ${\bar{F}}^{B}$ for $N_{C}$ times to enhance the features. Self-attention expands the receptive field, enabling the integration of the most relevant information for current feature updates, thereby making the key features more distinctive. Cross-attention allows the model to perceive similarities and differences between the two images, reducing noise from temporal-difference areas and obtaining a more robust feature representation. The coarse-scale feature maps after enhancement are denoted as $\bar{F}\prime^{A}$ and $\bar{F}\prime^{B}$. Assuming $\bar{F}\prime^{A},\bar{F}\prime^{B}\in R^{\bar{C}\times \bar{H}\times \bar{W}}$, in order to measure the similarity, they are flattened and the similarity matrix $S_{C}$ is constructed using the dot product:

(13) $$S_{C}\left(i,j\right)=<\bar{F}\prime^{A}\left(i\right),\bar{F}\prime^{B}\left(j\right)>,\forall i,j\in \left(0,\bar{H}\times \bar{W}\right]$$

in which $\bar{F}\prime^{A}\left(i\right)$ represents the *i*-th feature after $\bar{F}\prime^{A}$ is flattened, and $\bar{F}\prime^{B}\left(j\right)$ represents the *j*-th feature in $\bar{F}\prime^{B}$. <·,·> denotes the dot product operation. The softmax function is applied to both the rows and columns of $S_{C}$ to derive the matching probability matrix, which can be expressed as follows:

(14) $$P_{C}\left(i,j\right)={\mathrm{Softmax}}_{row=i}\left(S_{C}\right)\cdot {\mathrm{Softmax}}_{col=j}\left(S_{C}\right)$$

Matches with probabilities below the threshold $\theta_{C}$ are filtered and further refined using mutual nearest neighbor (MNN) criteria. The coarse-scale matches set $M_{C}$ is obtained.

### 3.3. Fine-Scale Matching

As shown in Figure 1, ${\tilde{F}}^{A}$ and ${\tilde{F}}^{B}$ are first updated by

(15) $$\begin{array}{c} {\tilde{F}}^{\dot{A}}=\mathrm{FC}\left(\left[{\tilde{F}}^{A}|\mathrm{Upsampling}\left(\mathrm{FC}\left(\bar{F}\prime^{A}\right)\right)\right]\right), \end{array}$$

(16) $$\begin{array}{c} {\tilde{F}}^{\dot{B}}=\mathrm{FC}\left(\left[{\tilde{F}}^{B}|\mathrm{Upsampling}\left(\mathrm{FC}\left(\bar{F}\prime^{B}\right)\right)\right]\right) \end{array}$$

in which [·|·] denotes the concentration and ${\tilde{F}}^{\dot{A}},{\tilde{F}}^{\dot{B}}\in R^{\tilde{C}\times \tilde{H}\times \tilde{W}}$. This update implicitly embeds the position encoding from the coarse scale into the fine scale, eliminating additional position encoding at this stage.

For every coarse match [${\bar{i}}_{c}$, ${\bar{j}}_{c}$] in $M_{c}$, the corresponding feature patches ${\tilde{F}}_{crop}^{A},{\tilde{F}}_{crop}^{B}\in R^{\tilde{C}\times w_{f}\times w_{f}}$ are cropped with a window size of $w_{f}\times w_{f}$ from ${\tilde{F}}^{\dot{A}}$ and ${\tilde{F}}^{\dot{B}}$. After obtaining a set of feature map patch pairs, the vanilla attention mechanism is employed to perform self-attention and cross-attention feature enhancement:

(17) $$\tilde{F}\prime_{crop}^{A}=\mathrm{CA}\left(\mathrm{SA}\left({\tilde{F}}_{crop}^{A}\right)\right),\tilde{F}\prime_{crop}^{B}=\mathrm{CA}\left(\mathrm{SA}\left({\tilde{F}}_{crop}^{B}\right)\right)$$

in which CA(·) denotes the vanilla cross-attention and SA(·) denotes the vanilla self-attention. Since the feature maps are cropped at this time, the computational complexity of the vanilla attention is within an acceptable range.

Then, $\tilde{F}\prime_{crop}^{A}$ and $\tilde{F}\prime_{crop}^{B}$ are flattened, and denote that $\tilde{F}\prime_{crop}^{A}$ is centered on the $\tilde{i}$-th vector. To locate the matching keypoints precisely, $\tilde{F}\prime_{crop}^{A}\left(\tilde{i}\right)$ is taken as the query and the similarity with the dot product of all vectors in $\tilde{F}\prime_{crop}^{B}$ is calculated:

(18) $$S_{F}\left(j\right)=<\tilde{F}\prime_{crop}^{A}\left(\tilde{i}\right),\tilde{F}\prime_{crop}^{B}\left(j\right)>,\forall j\in \left(0,w_{f}\times w_{f}\right]$$

A probability distribution can be obtained after applying softmax to $S_{F}$, which indicates the possibility that each point in $\bar{F}\prime_{crop}^{B}$ is the match point of $\tilde{F}\prime_{crop}^{A}\left(\tilde{i}\right)$:

(19) $$P_{F}\left(j\right)=\mathrm{Softmax}\left(S_{F}\left(j\right)\right)$$

By calculating the expectation of $P_{F}$, the final match $\tilde{j}\prime$ in $\bar{F}\prime_{crop}^{B}$ with sub-pixel accuracy on B can be obtained.

## 4. Experimental Results and Discussion

### 4.1. Experimental Setup

The experiments in this study were conducted on the Ubuntu 22.04 operating system using two NVIDIA GeForce RTX 3090 GPUs, each with 24 GB of memory. The Adam optimizer was used for the training, with an initial learning rate of 0.001. The learning rate was warmed up over the first three epochs, and a weight decay factor of 0.01 was applied. The momentum parameter was set to $\left[\beta_{1},\beta_{2}\right]=\left[0.9,0.999\right]$, and the batch size was 4. The training proceeded for 50 epochs. The loss function followed the settings used in LoFTR, which comprised a coarse-scale cross-entropy loss and a fine-scale Euclidean distance loss.

In the feature extraction stage, we tended to select $n_{f}=16$ groups of frequency components that were verified to be the most beneficial to feature extraction accuracy [43]. In the coarse-scale matching, we selected m=4 groups of the lowest frequency components, with the aim to balance the matching accuracy and computational efficiency. The window size was set to $w_{c}=8$ and the number of selected windows was k=8. We set $N_{C}=2$. $w_{c}$ was selected based on the input size during the model training. For different input sizes, we preferred to keep the number of windows consistent rather than the window size. The feature dimension remained $\bar{C}=256$ during this stage. In the fine-scale matching, the window size was $w_{f}=5$ and the feature dimension remained $\tilde{C}=128$.

### 4.2. Datasets

**Figure 4:**
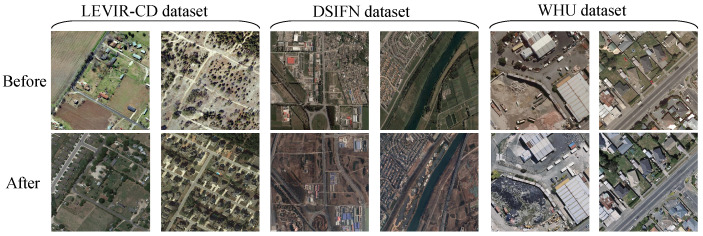
Examples of the datasets [51,52,53].

The three datasets used in this study were the LEVIR-CD dataset [51], the DSIFN dataset [52], and the WHU building change detection dataset [53] (Figure 4). The LEVIR-CD dataset contained 637 bitemporal image pairs with a time span of 5 to 14 years, of which 445 image pairs were used for training, 64 image pairs were used for validation, and 128 image pairs were used for testing. The image pair size was 1024 × 1024. The DSIFN dataset contained high-resolution bitemporal image pairs of six cities in China collected from Google Earth. Five cities were cropped into 394 sub-image pairs as training sets, and one city was cropped into 48 sub-image pairs as test sets. The WHU dataset consisted of two high-resolution (32,507 × 15,354) TIFF optical remote sensing images. The WHU dataset was only used for training purposes and not for testing. Specifically, we cropped the pair of images vertically with a width of 512 pixels to create the validation set, and used the remaining portion of the image for training. For the validation set, we applied non-overlapping 512 × 512 pixel windows to generate the validation patches. In contrast, for the training set, we cropped overlapping 512 × 512 pixel patches from the remaining part of the image, which helped create a more diverse and comprehensive set of training examples. The whole process ensured that both the original images were cropped at the same locations, and thus, formed the corresponding image pairs. We cropped all the images to size 512 × 512 and expanded them by mirror flipping and rotating them by 90°, 180°, and 270° to form the training datasets.

The specific steps to generate a single training instance were as follows: First, a pair of images $I_{A}$ and $I_{B}$ were cropped from the same position of two images from the initial dataset, with $I_{A}$ as image A. Then, $I_{B}$ was randomly translated, scaled, and rotated within a certain range to form image B, and the corresponding homography transformation matrix H was calculated. This resulted in A and B as a pair of cross-temporal remote sensing images, with H as the label. Additionally, random changes, such as adjustments in brightness and contrast, were applied to the images. In each round of training, the image pairs input into the model were randomly generated, which effectively prevented the model from overfitting during training.

### 4.3. Matching Experiment Evaluation Metrics

This study used the following metrics to evaluate the matching experiments: success rate (SR), percentage of correct keypoints (PCK), average corner error (ACE), number of trainable parameters (Params), and running time for one pair of images (RT). The images to be matched are denoted as *A* and *B*. The matching set of the image pair is $M=\{\left(k_{A},k_{B}\right)|k_{A}\in A,k_{B}\in B\}$. *H* is the homography transformation matrix obtained by applying the RANSAC [54] algorithm from *A* to *B*. The details of some metrics are as follows:

1. The success rate is calculated by
  (20) $$SR=\frac{N_{success}}{N_{total}},$$
  in which $N_{total}$ denotes the total number of image pairs in the dataset and $N_{success}$ denotes the number of successfully matched image pairs. We considered image pairs with more than 10 matching point pairs and an ACE of less than 100 pixels as successful matches.
2. The percentage of correct keypoints is calculated by
  (21) $$PCK=\frac{n_{correct}}{n_{total}},$$
  in which $n_{total}$ represents the total number of point pairs in *M* and $n_{correct}$ denote the number of correctly matched point pairs in *M*. The coordinates of the actual corresponding point of each $k_{A}$ in *B* are marked as $k_{B}^{gt}$. The Euclidean distance between $k_{B}^{gt}$ and $k_{B}$ is calculated. If this distance is less than the threshold ε (set to 3 pixels), the keypoint pair is considered correctly matched. PCK can be calculated for each pair of images, and the average PCK across all pairs is used for comparison.
3. The average corner error is calculated by
  (22) $$ACE=\frac{1}{N}\sum_{i=1}^{N}{∥c_{i}-c_{i}^{gt}∥}_{2},$$
  in which *N* denotes the number of corner points. We take the four vertices of the image as corner points, which means N=4. $c_{i}$ represents the position of the *i*-th corner point after the *H* transformation, and $c_{i}^{gt}$ represents the true position of the *i*-th corner point after transformation. We compared the ACE average values of the successfully matched image pairs.

### 4.4. Ablation Study and Discussion

**Figure 5:**
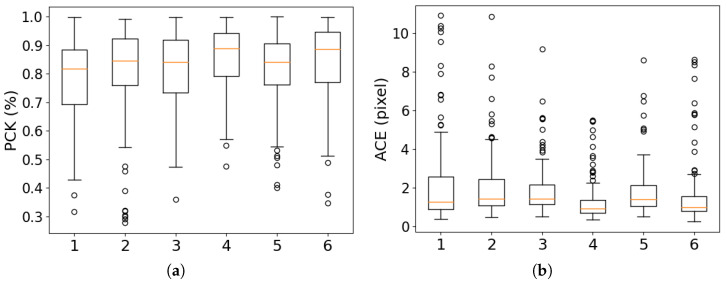
Box plots of ablation experiment results. The dots represent outliers of the results, and the orange lines indicate the median. (**a**) Box plot of PCK results. (**b**) Box plot of ACE results.

In order to prove the effectiveness of the eFCA in the backbone and the DSA in the coarse-scale matching module, we conducted ablation experiments. All models were trained on the same dataset, the optimizer settings were kept consistent, and the experimental results were measured on our validation set. The network without the eFCA and DSA was regarded as the baseline. Two attention mechanisms were added to the baseline and compared with our network (Experiment 4). The results are shown in Table 1, and more details are shown as box plots in Figure 5.

As shown in the table, the matching performance of the model was significantly influenced by these modules. The box plots show that the result distribution became notably more concentrated after incorporating attention mechanisms, indicating enhanced robustness and improved accuracy. It is evident that using frequency domain information to generate channel attention in multi-temporal remote sensing image matching contributed to obtaining richer detailed features. These two mechanisms effectively improved the accuracy. Overall, the DSA module had a greater impact on the matching effect than the eFCA module. The eFCA refined the importance of different feature channels by emphasizing relevant frequency components and suppressing irrelevant ones, which laid a solid foundation for the DSA to generate frequency-based attention guidance. The DSA used this frequency guidance to apply window attention, which enhanced the quality of the feature representation. In summary, the eFCA enhanced the quality of the feature extraction and overall feature representation, while the DSA further refined the feature representation by focusing on the most relevant regions of the image. Together, these modules improved both the global feature representation and local feature accuracy, ultimately boosting the model’s performance in image matching. Therefore, we believe that although the eFCA and DSA work independently on different stages, their combined use creates a synergistic effect.

In addition, we also compared the performance of the eFCA module with the original FCA module from the FCANet [43] article. The experimental results are presented in Table 2. As shown in this table, our method with the eFCA module significantly outperformed the one with original FCA module, particularly in terms of the ACE. Specifically, Experiment 4 achieved a reduction of 1.5 pixels compared with Experiment 5. This improvement can be attributed to the integration of richer frequency information by the eFCA. This enhancement allowed the model to better focus on key features, and thus, significantly improved the overall performance.

Furthermore, we also conducted comparative experiments between the DSA and the vanilla attention, and the results are shown in Table 3. Adding attention to the coarse-scale matching module significantly increased the trainable parameters. Compared with Experiment 2, the trainable parameters of Experiment 6 were almost doubled, but the attention brought about a greater performance improvement. The SR of Experiment 6 was 0.77 pixels higher than that of Experiment 4, but the comparisons of PCK and ACE were 1.88% and 0.43 pixels lower than Experiment 4, with a significantly longer inference time. The distribution of the results of Experiment 6, as shown in Figure 5, was more dispersed compared with Experiment 4. The experimental results verified that the global interaction of the features of the vanilla attention mechanism could improve the robustness of the model in complex scenes, but also introduced a large amount of redundant calculations. This led to an increased inference time and increased interference in the temporal difference areas during the attention allocation process, which negatively impacted the final accuracy of the model. In contrast, the DSA used frequency information to perceive the overall situation in advance and focused on regions with high feature similarity for the attention interaction, which greatly reduced the computational complexity and improved both the efficiency and practicality of the model.

### 4.5. Comparative Experiments and Discussion

In this section, we compare our methods with two categories of existing methods. The first category included matching methods based on manually designed features. We selected two prominent algorithms proposed in recent years: RIFT [12] and OFM [14]. The second category consisted of an end-to-end matching method based on deep learning. Super (SuperPoint [26] + SuperGlue [3]), LoFTR [4], and DeDoDe [55] were selected here. All methods were evaluated using the publicly available code and optimal parameter settings provided by the respective authors. The dataset and training settings were consistent with those used in this study.

#### 4.5.1. Quantitative Comparison

**Figure 6:**
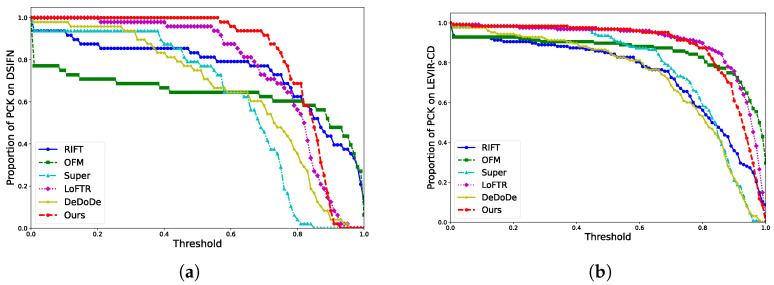
The line charts show the proportion of results that met different PCK thresholds. The x-axis represents the set PCK thresholds, and the y-axis represents the proportion of images in the dataset that satisfied each threshold. (**a**) Results on the DSIFN dataset. (**b**) Results on the LEVIR-CD dataset.

Table 4 presents the quantitative evaluation results of the six algorithms on the DSIFN dataset. Figure 6a shows the proportion of images in the dataset that met different thresholds of the PCK. In the evaluation on the DSIFN dataset, the method proposed in this paper outperformed all the other algorithms in terms of the SR and matching accuracy. In comparison, the SR of the manually designed methods was lower than that of the deep learning-based methods, indicating that the scene robustness of the manually designed method was limited. This limitation arose because these methods rely on human prior knowledge and rules. When the changes in the image exceeded expected ranges, the manual methods often failed to match or were inaccurate. However, in their applicable scenarios, they could achieve impressive matching accuracies. The line chart demonstrates that in some images, the accuracy of the detected keypoints approached 1, a result that was challenging for the deep learning-based methods to achieve. Among the deep learning methods, the method proposed in this paper was the best. When all the images in the dataset were successfully matched, the average PCK was 81.92%, which was 5.19% ahead of the second-best approach. The average ACE after the homography was only 4.27 pixels, which was 2.46 pixels better than the second-best approach. In addition, it is worth noting that our method had the fewest trainable parameters and the fastest running speed.

Table 5 presents the quantitative evaluation results of the six algorithms on the LEVIR-CD dataset, while Figure 6b illustrates the detailed results of the PCK. From the perspective of the SR, all algorithms performed better on the LEVIR-CD dataset than the DSIFN dataset. This was because most image pairs in the LEVIR-CD dataset had prominent and invariant geometric structures (e.g., roads) that facilitated matching, whereas the DSIFN dataset had a larger element size and contained more significant variations in the ground objects, which made matching more challenging. In the comparison on the LEVIR-CD dataset, our method achieved the highest matching success rate. Although its PCK was 1.93% lower than LoFTR, and the ACE was 0.94 pixels higher, our method outperformed LoFTR in terms of the inference speed. The slight differences in the PCK and ACE could be attributed to the trade-off between the accuracy and computational efficiency. Our method was designed for scenes with temporal differences and adopted a DCT-guided sparse attention mechanism to enhance the computational efficiency while reducing the interference in temporal regions. The greater the difference in the ground objects, the more pronounced the advantage of our method, as confirmed by the comparison on the DSIFN dataset. In scenes with fewer temporal changes, the amount of interference information naturally decreased, and more feature interactions tended to lead to a higher accuracy. However, the limited number of attention windows in our method may constrain its performance in such cases. In contrast, LoFTR had a more complex network structure, particularly its global attention mechanism, which allowed it to capture more information. As a result, LoFTR achieved a slightly higher accuracy in scenes with fewer temporal changes between images. Nevertheless, the advantages of our method in scene adaptability and faster inference speed make it a more practical solution for applications that require high efficiency and scalability. In terms of the running time, the proposed method was 25.96 ms slower than Super. This was because our method is a dense matching method, and the running time is influenced by the regional similarity. While the computational efficiency was less prominent than that in DSIFN, our method remained the second fastest algorithm among all the compared methods on LEVIR-CD.

**Figure 7:**
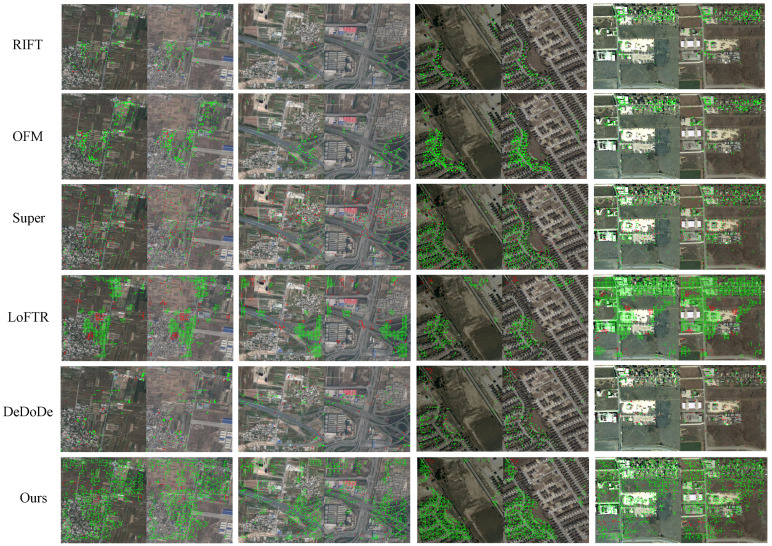
Visualization of some results from the DSIFN dataset and the LEVIR-CD dataset. The two groups on the left were from the DSIFN dataset, while the two groups on the right were from the LEVIR-CD dataset. The green dots indicate correctly matched keypoints, and the red dots indicate incorrectly matched keypoints.

Figure 7 visualizes some of the quantitative results. It can be observed that the keypoints detected by the manually designed methods were significantly clustered. This clustering could lead to overfitting in these regions when calculating the homography transformation, which resulted in poor adaptability to other regions. This explains why the ACE of the manually designed method was low. In contrast, the keypoints detected by the deep learning-based methods were more dispersed, which enhanced their ability to handle complex and changing scenes, which made it more robust. This also explains why the SRs of the deep learning-based methods were higher than those of the manually designed methods. In addition, the keypoints detected by our method could effectively avoid the temporal difference area. Considering the results across the two datasets, our method achieved the highest SR, indicating superior robustness in multi-temporal scenarios. The high PCK and low ACE further demonstrated that our method provided accurate matching while maintaining a high reliability in successful matching. Our method was also lightweight and fast to infer.

#### 4.5.2. Qualitative Comparison

**Figure 8:**
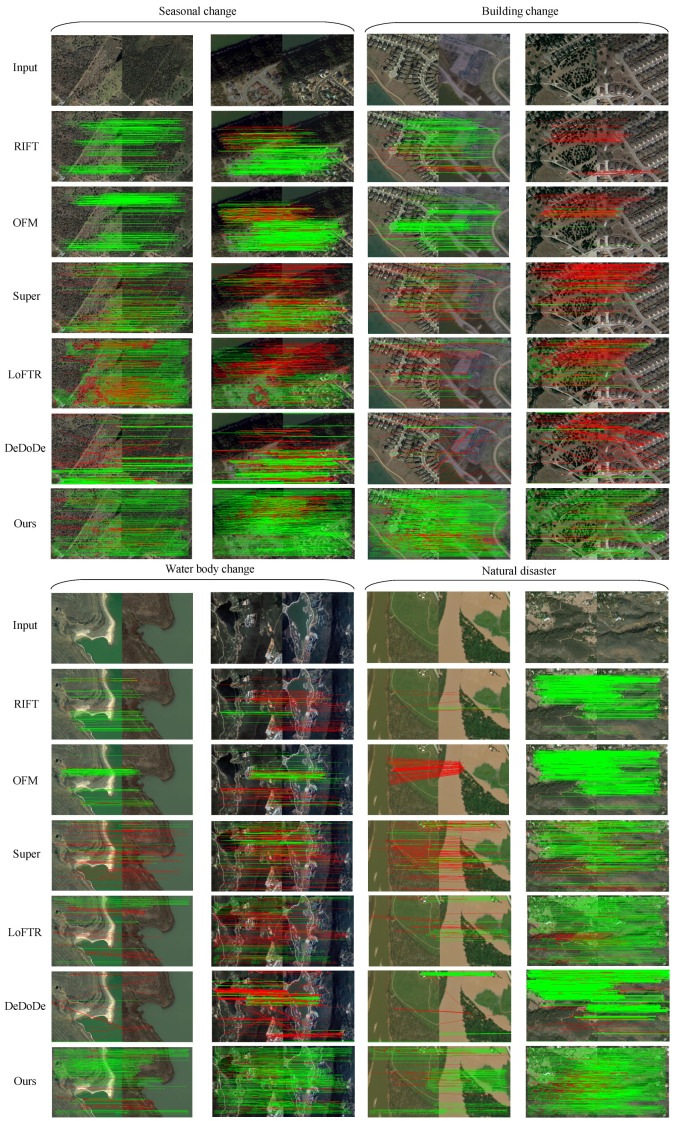
This is a visualization of qualitative comparison, with correct matching keypoints represented in green and incorrect matching keypoints represented in red.

To provide a comprehensive evaluation, we selected several representative image pairs from different datasets and conducted a qualitative analysis of all the algorithms. The multi-temporal scene changes were categorized into four types: seasonal changes, building changes, water changes, and natural disaster changes. The matching results are visualized in Figure 8. As shown in the figure, the performances of the RIFT and OFM methods were largely similar. Both algorithms achieved a high accuracy in scenes with minor changes, but they exhibited significant deviations in scenarios with large-scale building changes, water changes, or natural disasters (floods). Super could detect a certain number of matching points across all scenes, but it also produced more mismatches. LoFTR generally performed well in matching, though it struggled with large-scale building changes and water changes, which led to more mismatched points. DeDoDe, on the other hand, showed substantial errors and could only successfully match seasonal changes and natural disasters (forest fires). In contrast, our method effectively avoided regions with significant changes during the keypoint detection across all four scenes, which resulted in a higher accuracy. This qualitative analysis aligned with the quantitative results and validated the strong robustness and high precision of our proposed method.

## 5. Conclusions

To address the challenges posed by surface differences in multi-temporal remote sensing image matching, this paper proposes a remote sensing image matching network that combines a Discrete Cosine Transform (DCT) and attention mechanisms for multi-scale feature matching. The aim was to improve the robustness and accuracy of image matching in multi-temporal scenarios. The innovation of this network lies primarily in the enhanced frequency channel attention mechanism and the frequency-guided sparse attention mechanism. The former is used for multi-scale feature extraction, where the DCT is employed to integrate the frequency domain information of the feature map, forming weights that enhance the expression of key information in the feature map. The latter is used for coarse-scale matching, where the DCT transforms the feature map to generate attention guides, suppressing noise caused by temporal variations while reducing the computational burden. The experimental results show that our method outperformed the existing methods in terms of the inference speed and accuracy, and was more suitable for matching multi-temporal remote sensing images. This study mainly focused on the significant differences in ground objects between multi-temporal remote sensing images. However, the differences in multi-temporal scenarios may arise not only from changes in ground objects but also from day–night variations and sensor differences. In future work, we will explore the impacts of these additional factors to gain a deeper understanding of the task, enrich the dataset, and further improve the model structure to better address these challenges.

## Figures and Tables

**Table 1:** Ablation experiment evaluation results.

No	Approach	Params	SR (%) ↑	PCK > 80 (%) ↑	ACE (pix) ↓
1	Baseline	4.09 M	89.06	83.33	6.92
2	Baseline + eFCA	4.10 M	93.75	89.94	5.25
3	Baseline + DSA	5.69 M	96.09	85.76	4.08
4	Baseline + eFCA + DSA (ours)	5.70 M	**98.44**	**94.44**	**2.23**

↑: A higher metric value indicates better performance. ↓: A lower metric value indicates better performance.

**Table 2:** Comparison of different channel attention effects.

No	Approach	Params	SR (%) ↑	PCK > 80 (%) ↑	ACE (pix) ↓
4	Baseline + eFCA + DSA (ours)	5.70 M	**98.44**	**94.44**	**2.23**
5	Baseline + FCA + DSA	5.70 M	96.86	90.58	3.73

↑: A higher metric value indicates better performance. ↓: A lower metric value indicates better performance.

**Table 3:** Comparison of different attentions in coarse-scale matching effects.

No	Approach	Params	SR (%) ↑	PCK > 80 (%) ↑	ACE (pix) ↓	RT (ms) ↓
4	Baseline + eFCA + DSA (ours)	5.70 M	98.44	**94.44**	**2.23**	**66.39**
6	Baseline + eFCA + VanillaAttn	8.05 M	**99.21**	92.56	2.66	108.24

↑: A higher metric value indicates better performance. ↓: A lower metric value indicates better performance.

**Table 4:** Results of comparisons on DSIFN datasets.

Approach	Params	SR (%) ↑	PCK (%) ↑	ACE (pix) ↓	RT (ms) ↓
RIFT	-	87.50	72.03	13.41	-
OFM	-	70.83	62.03	10.62	-
Super	13.32 M	89.58	58.05	11.14	91.43
LoFTR	11.56 M	97.92	76.73	6.73	193.05
DeDoDe	13.52 M	85.42	64.87	10.67	155.72
Ours	5.70 M	**100**	**81.92**	**4.27**	**70.90**

↑: A higher metric value indicates better performance. ↓: A lower metric value indicates better performance.

**Table 5:** Results of comparisons on LEVIR-CD dataset.

Approach	Params	SR (%) ↑	PCK (%) ↑	ACE (pix) ↓	RT (ms) ↓
RIFT	-	90.63	70.47	10.48	-
OFM	-	92.18	83.72	5.92	-
Super	13.32 M	98.44	75.95	5.78	**172.43**
LoFTR	11.56 M	98.44	**90.41**	**2.04**	656.35
DeDoDe	13.52 M	89.06	72.36	8.27	406.30
Ours	5.70 M	**99.22**	88.48	2.98	198.39

↑: A higher metric value indicates better performance. ↓: A lower metric value indicates better performance.

## Data Availability

The data that support the findings of this study are available from the corresponding author upon reasonable request.
